# Non-Invasive Predictors of Response to Tamsulosin for Benign Prostatic Obstruction

**DOI:** 10.7759/cureus.13341

**Published:** 2021-02-15

**Authors:** Mohammad Shoaib, Muhibullah Bangash, Wajahat Aziz, M Hammad Ather

**Affiliations:** 1 Section of Urology, Department of Surgery, Aga Khan University Hospital, Karachi, PAK; 2 Section of Urology, Department of Surgery, Aga Khan University, Karachi, PAK

**Keywords:** benign prostatic obstruction, alpha-blockers, predictors

## Abstract

Objectives

To identify non-invasive predictors of response to tamsulosin 0.4 mg in patients with benign prostatic obstruction (BPO).

Methods

Males ≥ 50 years of age with lower urinary tract symptoms (LUTS) suggestive of BPO for over three months were included in the study. We assessed change in the mean International Prostate Symptom Score (IPSS) and maximum flow rate (Qmax) after six weeks of medical therapy. Clinical and uroflowmetry parameters were compared between two groups of patients with >25% vs. <25% change in the IPSS after treatment. Pre- and post-treatment post-void residue (PVR), Qmax, and IPSS were compared by independent t-test, univariate/multivariate regression analysis.

Results

A total of 121 patients were included. At presentation, the mean prostate size was 35.7±12.2 grams and the mean IPSS was 16.3 ± 4.8. Improvement in the mean IPSS was 7.83, with more marked improvement in storage compared to voiding LUTS (5.26 vs. 2.57). Majority (58%) had a quality of life (QoL) score of 4-5 at presentation whereas after 6-weeks of medication (83.5%) had a QoL score of 0-2. Treatment failure was noted in 11 (9.1%) patients. IPSS was higher and Qmax was lower at the time of presentation in patients who had <25% improvement. However, the two groups were identical on the basis of demographic and other factors (BMI, age, prostate size, PVR).

Conclusion

Moderate LUTS secondary to BPO responds favourably to alpha-blocker (tamsulosin 0.4 mg) treatment. Uroflowmetry (UFM) parameters, that is, Qmax and IPSS are important factors in predicting short-term response to medical therapy.

## Introduction

Benign prostatic obstruction (BPO) is a progressive disease often observed in men over 50 years of age. Enlargement of the prostate does not affect all men in a similar fashion [1]. BPO leads to a deterioration in the quality of life by disturbing normal activities such as sleep, and may cause complications such as recurrent UTIs, bladder stones or acute urinary retention. The most commonly performed surgical intervention for BPO is transurethral resection of the prostate. However, it is associated with short and long term morbidity [2]. Currently, medical treatment with alpha-blockers, 5-alpha-reductase inhibitors (5-ARIs) either alone or in combination has become the first line of therapy. Alpha-blockers, act by relaxing the prostatic smooth muscle and improve urination, are most commonly used for BPO patients.

Clinical and investigational workup in the evaluation of benign prostatic enlargement, besides baseline workup, include the International Prostate Symptoms Score (IPSS), uroflowmetry and post-void residual (PVR) urine or urodynamic studies [3].

An interventional urodynamic study, in particular pressure flow study, is the gold standard in the diagnosis of BPO secondary to BPH. Predictors of response to therapy are useful in stratifying patients for first-line medical versus surgical treatment [4]. These predictors can be classified as invasive and non-invasive. In some men, the symptoms are difficult to manage with medical therapy while others respond remarkably well to alpha-blockers + 5ARIs. This may be the result of different morphological characteristics of prostatic enlargement. Morphologically four types of prostatic enlargement are described such as isolated lateral or median lobe enlargement, lateral and middle lobe enlargement or posterior commissural hyperplasia [5].

There are several non-invasive tests available for the diagnosis of BPO in order to avoid the morbidity associated with invasive urodynamics. However, there is uncertainty concerning the diagnostic accuracy of these tests. Most of the guidelines endorse these tests as “mandatory” or “recommended” in the clinical evaluation of men with BPO. In a systematic review by Malde et al [6], authors noted high sensitivity and specificity of evaluation of detrusor wall thickness, near-infrared spectroscopy and the penile cuff test in diagnosing BPO.

We conducted this study to assess improvement in IPSS and determine predictors of urinary retention and the need for intervention in patients on medical treatment with alpha-blocker tamsulosin hydrochloride. Results of this study will help in counselling of management and the need for intervention.

## Materials and methods

We performed a cross-sectional study at the Department of Urology in a University Hospital. After ethical review committee approval, patients above 50 years of age presenting with lower urinary tract symptoms (LUTS) suggestive of BPO for more than three months were included. We excluded patients already on any medical therapy, patients with bladder stone, urinary tract infection, neurogenic bladder or urethral stricture. Patients who had an intervention for prostatic obstruction previously or who were unable to perform uroflowmetry (UFM) were also excluded. Data were collected over a period of nine months. Non-probability consecutive sampling technique was employed. The primary outcomes were improvement in mean IPSS and Qmax after six weeks. Secondary outcomes were urinary retention or need for surgery. The IPSS consists of seven questions that deal with voiding symptoms (incomplete emptying, intermittency, weak stream and straining to void) and storage symptoms (frequency, urgency and nocturia) in addition to another question, looking at the effect on the quality of life (QoL).

Data were analysed using SPSS version 25 (IBM Corp., Armonk, NY). Descriptive analyses for quantitative variables were reported as mean and SD, categorical variables such as basic demographic, clinical and other desired characteristics were reported as frequency and percentages. The pre- and post-treatment PVR, Qmax, and IPSS were reported as mean ± SD and were assessed by the independent t-test. P-value of less than 0.05 was considered statistically significant.

Response to medical therapy was seen in terms of improvement in IPSS at six weeks. Responders were defined as an overall improvement of ≥25% in IPSS score after six weeks. Predictors of response were identified with logistic regression analysis.

## Results

A total of 121 patients were included in our study. Mean age was 62.87 ± 8.78 and mean BMI was 26.05 ± 3.78. Thirty-five (29%) men were diabetic. The mean prostate size was 35.7g ± 12.2. Most patients (80%) had an IPSS of 8-18 (moderate score) on presentation.

In Table 1, the pre- and post-treatment parameters are described. Mean improvement in IPSS of 7.83 was noted after six weeks of tamsulosin, with more marked improvement in storage symptoms as compared to voiding symptoms (5.26 vs. 2.57). More than half of the patients (58%) had a QoL score of 4-5 (mostly unsatisfied/unhappy) at presentation whereas after six weeks of medication 83.5% had a QoL score of 0-2 (delighted/pleased/mostly satisfied). Treatment failure was noted in 11 (9.10%) patients. One patient developed urinary retention and the remaining 10 patients did not have any improvement in symptoms and were advised interventional treatment.

**Table 1:** Pre-treatment and post-treatment parameters. IPSS: International Prostate Symptoms Score; QoL: quality of life; PVR: post-void residue.

Variable	Pre-treatment	Post-treatment	P-value
IPSS	16.27 ± 4.78	8.45 ± 5.37	0.001
IPSS storage	9.28 ± 2.20	4.02 ± 2.48	0.001
IPSS voiding	7.0 ± 3.10	4.42 ± 3.11	0.001
QoL	4.14 ± 1.11	1.81 ± 1.16	0.001
PVR	77.09 ± 57.24	33.74 ±34.24	0.001
Qmax	10.41 ± 3.45	14.69 ± 5.53	0.001
Qavg	5.01 ± 3.45	8.02 ± 3.52	0.001

The group with >25% improvement in IPSS score was compared with those who had <25% improvement, no significant difference was found with regards to age, BMI and prostate size. However, a higher IPSS and PVR and lower Qmax and Qavg at the time of presentation were significant predictors of poorer response (<25% improvement in IPSS) (Table 2). On multivariate regression analysis, Qmax and IPSS at the time of presentation were found to be the only significant predictors of response to Tamsulosin (Table 3).

**Table 2:** Comparison of the non-invasive parameters in the groups. IPSS: International Prostate Symptoms Score; PVR: post-void residue.

	Not improved	Improved	P-value
	N = 18 (15%)	N = 103(85%)	
Age	63.50 ± 9.87	62.77 ± 8.52	0.67
BMI	25.81 ± 4.02	24.91 ± 3.74	0.84
Prostate size at presentation	35.36 ± 9.42	35.71 ± 12.68	0.55
PVR at presentation	106.71 ± 57.72	71.91 ± 55.83	0.27
IPSS storage	11.12 ± 2.09	8.97± 1.97	0.01
IPSS voiding	9.77 ± 4.14	6.50 ± 2.70	0.04
IPSS pre treatment	20.89 ± 5.93	15.47 ± 4.08	0.01
Quality of life	4.61 ± 1.20	3.70 ± 0.80	0.05
Qmax. at presentation	7.99 ± 3.29	10.82 ± 5.31	0.03
Avg. flow at presentation	3.48 ± 1.66	5.29 ± 1.72	0.04

**Table 3:** Univariate and multivariate regression analysis. IPSS: International Prostate Symptoms Score; QoL: quality of life.

Variable	Univariate analysis				Multivariate analysis			
	OR	95% CI		P-value	OR	95% CI		P-value
Post-void residue	0.96	0.96	0.99	0.02	0.99	0.98	1.00	0.66
Prostate volume	1.00	0.96	1.04	0.91				
Qmax.	1.51	1.84	1.94	0.01	1.28	1.01	1.62	0.03
QoL	0.34	0.19	0.62	0.01	0.98	0.41	2.33	0.96
IPSS	0.81	0.72	0.90	0.01	0.83	0.71	0.97	0.02

## Discussion

Lower urinary tract symptoms are a common presentation in a urological clinic. Significant proportion of these patients have BPO. Alpha-blockers are the mainstay of medical treatment along with lifestyle modifications. Men with detrusor hyperactivity respond better to combination treatment with α-blocker with an anticholinergic rather than monotherapy [7]. Combination treatment also makes use of PDE5 inhibitors (even in the absence of erectile dysfunction)[8] and 5 ARIs for larger gland sizes. Nevertheless, a significant proportion of these patients do not respond to the first-line medical treatment. There are multiple reasons for medical treatment failure and prediction of success is helpful in instituting the appropriate treatment on time.

In order to improve the efficacy of medical management, it is important to first and foremost determine whether these symptoms are due to BPO. The gold standard in the diagnosis of BPO is pressure-flow study [9]. However, it is invasive, cumbersome and expensive. Most current guidelines recommend the use of invasive urodynamic only in the select group of patients with equivocal outcome from non-invasive tests or in patients with associated medical conditions that could be the cause of underlying neurogenic bladder dysfunction [10]. Multiple studies have considered predictors of failure of medical therapy using non-invasive tests. These include intra-vesical prostatic protrusion, prostatic urethral angle (PUA), non-invasive urodynamic parameters, PVR, IPSS bladder wall thickness, etc [1,2,11,12,13].

However, in a clinical setting, only limited workup is available at initial presentation as recommended by most urological guidelines, for example, symptom score questionnaires, frequency volume chart (FVC), digital rectal examination(DRE) [14]. In the current study, we attempted to identify factors, which have the potential to predict response to medical therapy.

We found that IPSS at the time of presentation is significant when differentiating potential responders and non-responders to Tamsulosin treatment. However other studies showed that there is a poor correlation of IPSS to bladder outlet obstruction but patient reported symptoms in terms of IPSS do help in deciding appropriate management to some extent [15]. It was interesting to note that improvement in IPSS was less significant in voiding symptoms, which is similar to studies reporting a poor correlation of IPSS with voiding symptoms in patients with BPO [16,17]. We also found that Qmax at the time of presentation is also a significant predictor of response to tamsulosin treatment. In our study, Qmax less than 8 did not show improvement in IPSS and can be correlated with BPO. However, one study reported a Qmax < 4.5 mL/sec that was considered obstructed. Similarly, patients with Qmax of more than 11 ml/sec showed significant improvement in IPSS in our study in comparison to Qmax of 13.8 ml/sec reported in the literature [18]. In our study, prostate size and PVR are not significant predictors of poor response which is contrary to other studies reporting the prostate size and PVR [1,19] are significant predictors of failure of medical therapy. Although PVR alone cannot predict BPO as these patients do often have detrusor under-contractility [20,21] and the sensitivity and specificity of prostate volume as a predictor of BPO is very low [22].

In our study, only short-term response to medical therapy was assessed and in long-term factors found insignificant, may still be important. We only considered the clinically available workup for stratifying responders and did not include any interventional/investigational factors like urodynamics, which although ideal but usually not required in most patients presenting to a urology clinic. Results of this study will help patients in further deciding their treatment options at least in short term with the help of easily available, safe and quick non-invasive tools. It is a single-center study, so our results may not be applicable to patients who require more extensive workup, for example, those with the underlying neurological condition. 

## Conclusions

In the literature, extensive work has been done and most patients presenting with moderate LUTS secondary to benign prostatic obstruction respond favourably to alpha-blocker treatment. In our study, we also determined that UFM parameters specially Qmax and IPSS are important factors in predicting short-term response to medical therapy.
